# Clinical outcomes of three follitropin alfa preparations for ovarian stimulation using an oral micronized progesterone-primed protocol in an oocyte donation program

**DOI:** 10.3389/fendo.2024.1451668

**Published:** 2024-09-27

**Authors:** María Cruz, Colin M. Howles

**Affiliations:** ^1^ IVI-RMA Global Headquarters, Madrid, Spain; ^2^ ARIES Consulting Sàrl, Geneva, Switzerland; ^3^ Deanery of Biomedical Sciences, University of Edinburgh, Edinburgh, Scotland, United Kingdom

**Keywords:** follitropin alfa, medroxyprogesterone acetate, FSH biosimilar, ovarian stimulation, oocyte donation, ongoing pregnancy rate, cumulative live birth rate

## Abstract

**Introduction:**

This large multicenter study aimed to evaluate clinical outcomes using three follitropin alfa preparations within a progestin-primed ovarian stimulation (PPOS) protocol, while identifying contributing factors to cycle success.

**Methods:**

A retrospective, anonymized cohort analysis was conducted on donor-recipient cycles from 12 clinics during 2019 to 2021. 7389 oocyte donors underwent ovarian stimulation (OS) with three follitropin alfa preparations (Ovaleap^®^ [n=3231], Bemfola^®^ [n=3542], Gonal-F^®^ [n=616]) were included. Stimulation began on cycle days 2 or 3 with daily administration of 150-225 IU follitropin alfa. 10 mg medroxyprogesterone acetate (MPA) was administered daily until GnRH agonist trigger using a single dose of 0.2mg GnRH agonist for final follicular maturation. Statistical analysis included ANOVA, Chi-squared, and logistic regression.

**Results:**

Whilst there were some differences in patient and stimulation characteristics, including donor age and number of retrieved oocytes, clinical variables did not significantly differ among the three study groups. Linear regression revealed donor age [0.986 (0.974-0.999)] and number of mature oocytes [1.027 (1.007-1.047)] significantly impacted ongoing pregnancy rates, while the type of follitropin alfa [1.048 (0.956-1.149)] used did not. No significant differences were observed in the cumulative live birth rate (CLBR) among oocytes obtained from stimulation with Bemfola (64.9%), Gonal-F (64.1%) and Ovaleap (66.1%), p= 0.385.

**Discussion:**

This study demonstrated comparable clinical outcomes and CLBR between biosimilars and the reference product of follitropin alfa within PPOS protocols, hence they are interchangeable in a real-world patient setting.

## Introduction

Progestin-primed ovarian stimulation (PPOS) is an innovative approach used in Assisted Reproduction (IVF/ICSI cycles). This new strategy, based on the suppression of endogenous gonadotropins by progesterone during the luteal phase (1), has emerged as an efficient alternative to GnRH analogue antagonists (2) to suppress LH secretion and prevent an endogenous LH peak. The exact mechanism by which progesterone inhibits gonadotropin synthesis is still not fully understood; however, the most common hypothesis is that it is due to a hypothalamic effect; it has been reported the depletion of LH reserves in gonadotropic cells due to continuous exposure to progesterone (3, 4).

In recent years, the PPOS protocol has been utilized in different patient profiles undergoing OS (5). The studies published to date consistently report similar effectiveness and safety to that associated with GnRH analogues (6). Furthermore, the PPOS alternative provides an ovarian response comparable to that observed with GnRH antagonists; oral administration makes them much more patient-friendly, and when a fresh transfer is not required, it can become the more cost-effective option.

Gonadotropins used to stimulate follicular development and induce ovulation (7) are an essential component of the protocol to ensure the recruitment of multiple follicles and hence recovery of a cohort of oocytes. Today, in many parts of the world, there is a wide variety of gonadotropins available including biosimilar FSH medicines to the originator product follitropin alfa, thus expanding the possible treatment options for both healthcare professionals and patients. According to the European Medicines Agency (EMA), who were the first agency to define a regulatory pathway for approval, a biosimilar is a biological medicine that has been demonstrated through a series of physicochemical, *in vitro*, *in vivo* tests, and confirmatory Phase I and Phase III studies to be similar/equivalent in quality, safety, and efficacy to the reference medicinal product. Some other high performing regulatory agencies (termed WHO-listed Authorities, which includes those of US FDA, Japan, Canada, Australia, European Economic Area and UK MRHA), adhere to common guidelines on the evaluation of biosimilars and approval requirements (8, 9). While some post-approval data demonstrates that FSH biosimilars can be just as safe and effective as the original product in real-world settings (10, 11), there is still some confusion and conflicting findings on their safety and efficacy (12–14). Some of this confusion originates from a lack of understanding (15) of the rigorous registration process for a biosimilar, adopted by the European Medicines Agency and other WHO-listed Authorities (9). This is illustrated in a comprehensive analysis of the structural features of originator follitropin alfa versus so called “biosimilar follitropin alfa” preparations approved by non WHO-listed Authorities, including Russia, China, India, Mexico and Argentina (16). Here, Manzi and colleagues identified various physicochemical differences between reference follitropin alfa and the other FSH preparations, demonstrating non-biosimilarity. Thus, there is still a need for further studies to facilitate decision-making on adoption and switching to an FSH biosimilar from the originator molecule.

The PPOS protocol has gained attention in the context of egg donation cycles for Assisted Reproduction as the potential benefits of using this approach could also be applied to women seeking IVF fertility treatment. The objective of this study was to assess clinical outcomes using different follitropin alfa preparations in the context of a PPOS protocol.

## Material and methods

Multicenter, retrospective, anonymized cohort analysis in donor-recipient cycles conducted during 2019-2021 in 12 clinics belonging to the IVI-RMA group in Spain. Informed consent was not required because the study was based on non-identifiable records, as approved by the Ethics Committee (Institutional Review Approval 2006-MAD-048-AR). The study complied with the Spanish law governing assisted reproductive treatments (14/2006).

Oocyte donors were healthy women aged 18-35 years with regular menstrual cycles, a body mass index of 18-28 kg/m^2^, and no relevant medical history. They had a normal karyotype and fulfilled national legal requirements. The study population included women accepted as oocyte donors and undergoing OS with three different follitropin alfa preparations Ovaleap^®^ (Theramex Spain); Bemfola^®^ (Gedeon Richter Iberica); or Gonal-F^®^ (Merck SL). Ovaleap^®^ and Bemfola^®^ are both biosimilars, i.e. contain the same active pharmaceutical ingredient (follitropin alfa) to the originally approved biological product, Gonal-F^®^. Medical records of the egg donors who had undergone an OS protocol with any of these products were obtained from our clinical database (SIVIS, IVI Digital Information Management Platform). The recruited mature oocytes were donated to be used both in fresh and vitrified cycles, and treatment assignment was at the discretion of the clinician.

Ovarian stimulation was initiated on the second or third day of the menstrual cycle. The initial dose of recombinant FSH was 150-225 IU, depending on age, body mass index, and the results of ovarian reserve tests (17). Egg donors received either one of the three follitropin alfa products analyzed in this study. In all cases, from the first stimulation day, 10 mg of medroxyprogesterone acetate (MPA, Progevera, Pfizer, Spain) was administered orally as a single daily dose throughout stimulation until trigger day. Finally, a single subcutaneous bolus of 0.2 mg of triptorelin (Decapeptyl, Ipsen Pharma, France) was administered as soon as more than 3 follicles were 17 mm or larger, to trigger follicular and oocyte maturation. Transvaginal oocyte retrieval was performed 36 hours later.

Oocyte recipients were women aged under 50 years old who requested oocyte donation mainly owing to advanced maternal age. Endometrial preparation has been previously described (18). Approximately 10 days after initiating oral or transdermal estrogens, serum estradiol, and endometrial thickness were measured. Single embryo transfer at the blastocyst stage was performed in all the cases.

The main variable of the study was the ongoing pregnancy rate among the recipient of oocytes derived from one of these three study groups; this variable was defined as pregnancy documented by ultrasound at 12 gestational weeks that showed the presence of a fetal heartbeat in all cases where a blastocyst had been transferred.

Other clinical secondary outcome measures were defined as follows: the usable blastocyst rate as the number of transferred, frozen or biopsied blastocysts per the total number of fertilized oocytes; implantation rate as the number of fetal sacs identified by ultrasound of the total number transferred; clinical pregnancy rate as the number of pregnancies with confirmed ultrasound sacs out of the total number of embryo transfers; miscarriage rate as the number of positive fetal heartbeats lost from the total number of pregnancies; cumulative live birth, defined as the proportion of deliveries with at least one live birth per started cycle or oocyte retrieval, (including all fresh and/or frozen embryos transfers) until one newborn delivery or until all embryos were used, whichever occurred first.

Statistical analysis included mean ± standard deviation which was reported for continuous variables and percentages were applied for categorical variables. The Chi-squared test was used to evaluate associations among categorical associations while a Mann-Whitney test was performed to compare means between the different study groups. Finally, a logistic regression analysis was performed taking the ongoing pregnancy rate as the dependent variable. The significance level was set at p<0,05 and all the statistical analysis was performed with the Statistical Package for Social Sciences 23 (SPSS; Chicago, IL; USA).

## Results

In total, over the study period 2019-2021, 7389 donor-recipient cycles were included, analyzed and distributed as follows: 3231 received Ovaleap^®^ (Theramex Spain) for OS; 3542 were stimulated with Bemfola^®^ (Gedeon Richter Iberica); and n=616 were administered with the originator product, Gonal-F^®^ (Merck SL).

It was observed that there were some differences in donor demographics as well as stimulation outcomes between the 3 treatment groups (see **Table 1**). For example, donors stimulated with Gonal-F^®^ had significantly higher age and a longer stimulation phase, while donors stimulated with the FSH biosimilar Ovaleap^®^ had more retrieved and metaphase II oocytes. However as far as the total FSH dose required, oocyte donors stimulated with Gonal-F^®^ needed less FSH units to complete follicular development compared to Ovaleap^®^ or Bemfola^®^ (total FSH dose (IU) 1666 ± 50 vs. 1918 ± 40, vs 1911 ± 20 p<0.001) While these differences were significantly different, because of the large sample size and the homogeneity of the study population, these were however, not clinically relevant.

**Table 1:** Donor demographics and ovarian response variables for Bemfola^®^ vs Gonal-f^®^ vs. Ovaleap^®^ treatment.

	Bemfola^®^ (n=3542)	Gonal-f^®^ (n=616)	Ovaleap^®^ (n=3231)	p-value
Donor’s Age (years)	25.2 ± 0.2	25.3 ± 0.4	24.5 ± 0.3	<0.001
Donor’s weight (Kg)	61.1 ± 0.4	62.6 ± 0.8	60.8 ± 0.5	0.005
AMH (ng/ml)	2.8 ± 1.1	3.2 ± 0.6	3.1 ± 1.2	0.001
AFC	22.8 ± 0.3	21.7 ± 0.6	24.0 ± 0.5	<0.001
Days of FSH stimulation	10.2 ± 0.7	10.8 ± 0.5	10.1 ± 0.1	0.004
Estradiol_day of hCG (pg/ml)	3392 ± 135	3841 ± 360	3505 ± 150	0.126
Progesterone_day of hCG (ng/ml)	1.3 ± 0.1	0.9 ± 0.1	1.1 ± 0.1	0.675
Total Dose FSH (IU)	1911 ± 20	1666 ± 50	1918 ± 40	<0.001
Cancellation rate (%)	3.2%	4.1%	2.7%	<0.001
Oocytes retrieved	23.6 ± 0.5	23.9 ± 1.2	25.0 ± 1.1	<0.001
Oocytes metaphase II	19.7 ± 0.6	20.5 ± 0.9	22.1 ± 1.5	<0.001

(Mean values ± SD or %).

Regarding the clinical outcomes in the recipient population (**Table 2**), no significant differences were observed among the three study groups in pregnancy or miscarriage outcomes. Clinical outcomes for the Bemfola^®^, Gonal-F^®^ and Ovaleap^®^ derived oocytes were as follows; implantation rate 59.7% vs. 60.5% vs. 62.1%, p= 0.524; miscarriage rate 8.9% vs. 8.4% vs. 7.9%, p= 0.454; and ongoing pregnancy rate 51.7% vs. 53.4% vs. 56.1%, p= 0.321, respectively.

**Table 2:** Recipient demographics and clinical outcomes for Bemfola^®^ vs Gonal-f^®^ vs. Ovaleap^®^ treatment (Mean values ± SD or %).

	Bemfola^®^ (n=3542)	Gonal-f^®^ (n=616)	Ovaleap^®^ (n=3231)	p-value
Recipient’s age	42.3 ± 0.2	41.7 ± 0.4	42.8 ± 0.3	<0.001
Recipient’s BMI (kg/m^2)^	22.3 ± 0.4	23.1 ± 0.8	22.7 ± 0.5	0.012
Fertilization rate (%)	78.7%	79.7%	78.9%	0.065
Frozen embryos	4.0 ± 0.2	4.1 ± 0.3	3.9 ± 0.2	0.049
Implantation rate (%)	59.7%	60.5%	62.1%	0.524
Pregnancy rate (%)	60.8%	61.1%	62.5%	0.519
Miscarriage rate (%)	8.9%	8.4%	7.9%	0.454
Ongoing pregnancy rate (%)	51.7%	53.4%	56.1%	0.321
Cumulative livebirth rate (%)	64.9%	64.1%	66.1%	0.385

These findings were further confirmed by applying a linear regression model (**Table 3**), where the age of the donor [0.986 (0.974-0.999)] and the number of mature oocytes [1.027 (1.007-1.047)] significantly affected the chances of achieving an ongoing clinical pregnancy while the type of follitropin alfa [1.048 (0.956-1.149)] did not influence the success rate.

**Table 3:** Linear regression analysis for ongoing pregnancy rate.

Variable	OR (CI 95%)	P Value
Donor’s Age	0.986 (0.974-0.999)	0.033
Donor’s weight	1.002 (0.996-1.007)	0.531
Retrieved oocytes	0.999 (0.994-1.004)	0.414
Metaphase II oocytes	1.027 (1.007-1.047)	0.008
Type of follitropin alfa	1.048 (0.956-1.149)	0.317

Finally, and because the cumulative live birth rate (CLBR) is a crucial and comprehensive measure in assessing the success of an Assisted Reproductive treatment, no significant differences were observed in this parameter among oocytes obtained from stimulation with Bemfola^®^ (64.9%), Gonal-F^®^ (64.1%) and Ovaleap^®^ (66.1%), p= 0.385. Thus, it can be concluded that regardless of the type of follitropin alfa used for ovarian stimulation in a PPOS-protocol, the probability of treatment success is similar.

## Discussion

Laboratory, clinical and drug delivery advances in Assisted Reproductive treatments have improved outcome results and personalized specific procedures that a patient need (19). However, there is still further work required to improve OS protocols in terms of efficacy, safety, and patient convenience. In this context, the PPOS emerges as a feasible alternative that have shown similar results to previous GnRH analog protocols, in addition to being a more economical, simple, and patient-friendly option.

As far as we know, this is the first and largest study that analyzed clinical outcomes of follitropin alfa originator and its two biosimilars in donor-recipient cycles where a PPOS protocol had been employed. A wide range of progestins have already proven successful and safe in an extensive variety of freeze-all cycles (20–22). The PPOS strategy has also generated great interest in the field of oocyte donation, since a high number of metaphase II oocytes can be obtained with fewer injections overall, and therefore, with improved convenience for the egg donor (17, 23).

While there was a significant difference in the donor age between treatment groups (mean ranging from 24.5 to 25.3 years), it was not deemed clinically relevant and significant changes in fertility cannot be inferred. Oocyte donation is usually considered the last resort to achieve a pregnancy which many women undergoing fertility treatment desire. According to the results of this retrospective study, recipients from donors stimulated with any of the three follitropin alfa preparations approved by one of the WHO-listed Authorities have similar clinical outcomes in terms of ongoing pregnancy and cumulative live birth rate when used PPOS protocol.

During a medical consultation, it is relevant from the physician’s and patient’s perspective to consider all available evidence to evaluate whether there are clinically significant differences in quality, safety, and reproductive outcome after using biosimilar preparations compared to the reference product. The EMA introduced guidelines on biosimilar development in 2004 and specific recombinant FSH biosimilar guidelines in 2013. Since the launch of the first biosimilar in 2006, the EMA approved to date, a total of 93 biosimilars, and the totality of evidence acquired to date suggest that these biosimilars can be used as safely and effectively in their approved indications as other biological medicines (24). Despite this reassurance from a central regulatory body, there is still some ongoing confusion within the reproductive medicine community on what a biosimilar is, leading to resistance to its use. The suggested differences reported in early meta-analyses and associated reviews between biosimilars, and the originator seem to revolve around subtle variations in the FSH isoform (glycan) composition (12, 14), which are minor compared to those between follitropin alfa and beta (25). Additionally, each manufactured batch of every biological medicine will have inherent slight differences in glycosylation, but these differences are under strict control (26), which is a prerequisite to delivering a guaranteed dose of FSH from batch to batch. These issues have been comprehensively reviewed in a series of editorials and commentaries (27, 28) with the conclusion that FSH biosimilars can be used as safety and effectively in their approved indications as the originator biological medicine. However, a further retrospective analysis from Grynberg et al. (13), on data from 2013-2018, using the SNDS (French payments database) between follitropin alfa, FSH biosimilars, and HMG also suggests differences in outcomes not only for the biosimilars but also with HMG to the originator. Our findings, however, demonstrate that ongoing pregnancy and the cumulative live birth rate were similar with all follitropin alfa products and extend the findings of previously published studies of clinical outcomes between an FSH biosimilar and the originator as well as with follitropin beta (10, 11, 29, 30).

Gonadotrophins play a critical role in follicle recruitment, demonstrated by the relationship between oocytes retrieved and ongoing pregnancy and live birth rates (29–35). In this study, all three products yielded between 24-25 oocytes per stimulation. Cumulative live birth rate per OS has been increasingly identified as the standard clinical approach to measure the success of an Assisted Reproductive treatment (36). The results of the linear regression, which identified age of the donor and the number of oocytes retrieved as being significantly correlated with ongoing pregnancy, agree with the conclusions of a comprehensive review on which covariates are the most important to predict cumulative live birth (37). They also concluded that type of gonadotrophin was not found to be a covariate of value in any of the models that they reviewed.

To summarize, our analysis demonstrated that there were no significant differences among the three study groups in the cumulative live birth rate. This endpoint is the cumulation of numerous steps in the IVF procedure, from initial consultation, OS to oocyte recovery, *in vitro* embryo development, transfer, and luteal phase support. Numerous factors other than the result of OS are critical for a successful pregnancy and live birth (38).

The main limitations of the study are its retrospective nature and the population that underwent stimulation were young, with a normal ovarian response to gonadotropin treatment. This does not reflect the actual IVF patients treated in infertility clinics. However, as far as gaining information on the ability of these three follitropin alfa products to stimulate follicle development and ultimately yield similar cumulative live birth rates, this adds important information to the literature.

## Conclusions

This large multicenter retrospective anonymized cohort analysis was conducted on donor-recipient cycles from 12 IVF centers. There were no significant differences in clinical pregnancy outcomes between the three follitropin alfa preparations when used in the PPOS protocol. Small but controlled differences in glycosylation to the originator do not have any impact on clinical results, suggesting that different brands of follitropin alfa, registered by WHO-listed Authorities, are interchangeable in a PPOS stimulation protocol in a real-world patient setting.

## Data Availability

The raw data supporting the conclusions of this article will be made available by the authors, without undue reservation.
